# Draft Genome Sequence of Broad-Spectrum Antifungal Bacterium *Burkholderia gladioli* Strain NGJ1, Isolated from Healthy Rice Seeds

**DOI:** 10.1128/genomeA.00803-15

**Published:** 2015-07-23

**Authors:** Gopaljee Jha, Isha Tyagi, Rajeev Kumar, Srayan Ghosh

**Affiliations:** National Institute of Plant Genome Research, Aruna Asaf Ali Marg, New Delhi, India

## Abstract

We report here the draft genome sequence of *Burkholderia gladioli* strain NGJ1. The strain was isolated from healthy rice seeds and exhibits broad-spectrum antifungal activity against several agriculturally important pathogens, including *Rhizoctonia solani*, *Magnaporthe oryzae*, *Venturia inaequalis*, and *Fusarium oxysporum*.

## GENOME ANNOUNCEMENT

*Burkholderia* spp. are rod-shaped Gram-negative bacteria found in diverse habitats. Some of them are reported to be pathogenic to plants and humans; however, a few plant growth-promoting, endophytic, and antifungal strains with potential biocontrol activity have also been reported (1–5). We have isolated a yellow-pigmented bacterium from surface-sterilized healthy IR64 rice seeds, which demonstrated broad-spectrum antifungal activity against *Rhizoctonia solani* (the causal of sheath blight disease of rice), *Magnaporthe oryzae* (the rice blast pathogen), *Venturia inaequalis* (the apple scab pathogen), *Fusarium oxysporum* (vascular wilt pathogen), and several other fungal pathogens. 16S rRNA gene sequencing, deep phylogeny, and segregation rooting analyses identified the bacterium to be *B. gladioli*, and henceforth, the strain was named *B. gladioli* NGJ1.

The draft genome sequence of the bacterium was determined using the Illumina NextSeq 500 sequencing system with a paired-end library. A total of 36,488,466 paired-end reads were produced, and after quality trimming and error correction, 33,951,148 (93.05%) high-quality reads were retained. Sequence processing and assembly were performed using the A5 assembly pipeline (version A5-miseq 20150522), according to the workflow described by Tritt et al. (6). The assembly resulted in 155 contigs (minimum, 503 bp; maximum, 573,482 bp; *N*_50_, 283,251 bp). The final assembly contained 8,020,595 bp, with a G+C content of 68.07% and a median coverage of 650×. Genome completeness was assessed using the AMPHORA software version 2 (7, 8), which reflected the presence of 31 highly conserved phylogenetic marker genes in the draft genome of the bacterium. Interestingly, some of these marker genes had less-confident assignment scores to *B. gladioli*, suggesting divergence of the NGJ1 strain from other sequenced strains of *B. gladioli*. Annotation using Prokka version 1.11 (9) predicted 6,865 protein-coding sequences, 68 tRNAs, and 1 transfer-messenger RNA (tmRNA) in *B. gladioli* NGJ1. The genome is enriched in genes encoding secondary metabolites, microbial metabolism in diverse environments, biosynthesis of antibiotics, etc. This correlates with its antifungal ability and indicates its adaptability in diverse habitats. The detailed comparative and functional genomic analyses of this bacterium will help identify novel antifungal compounds and reveal their potential utilization in managing plant diseases.

### Nucleotide sequence accession numbers.

This whole-genome shotgun project has been deposited at DDBJ/EMBL/GenBank under the accession no. LEKY00000000. The version described in this paper is version LEKY01000000.
